# The North-Eastern Hospital for Children

**Published:** 1908-02-08

**Authors:** G. Edmonds, George Billings, H. Busby Bird, W. S. Wright

**Affiliations:** Mayor of Bethnal Green.; Mayor of Hackney.; Mayor of Shoreditch.; Mayor of Stoke Newington.


### THE NORTH-EASTERN HOSPITAL FOR CHILDREN.

Sir,—Being closely in touch with the local life of the districts surrounding the North-Eastern Hospital for Children, Hackney Road, Bethnal Green, we feel called upon to draw the attention of the public to the position of urgent need in which that institution now stands. In the maintenance of 130 beds and the treatment of some 28,000 children as out-patients annually the hospital spends nearly £11,000 a year, while its assured income, apart from charitable contributions, is under £1,000. Having started 1908 £2,000 in arrear, and having, in addition, a mortgage of £6,250 at 4 per cent. interest on the buildings, the committee feel great anxiety as to the immediate future, as it is evident that the hospital must shortly be in very serious difficulties unless substantial donations are forthcoming. Great efforts are being made in these districts to give the much-needed helping hand, but our people, though readily subscribing their shillings and pence whenever possible, are far too poor to accomplish the task unaided, and we ask the section of the public that is able to do so (but few of whom now make their homes in our midst) to come forward liberally with funds for our Children's Hospital at this critical period, and so to have the satisfaction of helping those who are doing their best to help themselves. Cheques, etc., crossed "Barclay's, Lombard Street," should be sent to the Secretary, Mr. T. Glenton-Kerr, at the hospital, and it may be added that contributions of whatever amount, large or small, will be gratefully appreciated.

We are sir, your obedient servants,

G. Edmonds, Mayor of Bethnal Green.
George Billings, L.C.C., Mayor of Hackney.
H. Busby Bird, Mayor of Shoreditch.
W. S. Wright, Mayor of Stoke Newington.

